# Effect of a Multispecies Probiotic on Intestinal and Skin Colonization by Multidrug-Resistant Gram-Negative Bacteria in Patients in a Long-Term Care Facility: A Pilot Study

**DOI:** 10.3390/nu12061586

**Published:** 2020-05-28

**Authors:** Ines Zollner-Schwetz, Monika Scarpatetti, Gerald Pichler, Christian Pux, Ingeborg Klymiuk, Slave Trajanoski, Robert Krause

**Affiliations:** 1 Section of Infectious Diseases and Tropical Medicine, Department of Internal Medicine, Medical University of Graz, Auenbruggerplatz 15, 8036 Graz, Austria; robert.krause@medunigraz.at; 2 Geriatric Health Centres of the City of Graz, Albert-Schweitzer-Gasse 36, 8020 Graz, Austria; monika.scarpatetti@stadt.graz.at (M.S.); gerald.pichler@stadt.graz.at (G.P.); christian.pux@stadt.graz.at (C.P.); 3 Core Facility Molecular Biology, Centre for Medical Research, Medical University of Graz, Stiftingtalstraße 24/1, 8010 Graz, Austria; ingeborg.klymiuk@medunigraz.at (I.K.); slave.trajanoski@medunigraz.at (S.T.)

**Keywords:** bacterial resistance, eradication treatment, intestinal decolonization, probiotics, long-term care facility

## Abstract

Residents in long-term care facilities (LTCFs) are frequently colonized by multidrug-resistant Gram-negative bacteria, putting them at risk for subsequent infections. We aimed to evaluate the effect of the multispecies probiotic Omnibiotic10AAD^®^ on the intestinal and inguinal skin colonization of patients by multidrug-resistant Gram-negative bacteria in LTCFs. Patients colonized by multidrug-resistant Gram-negative bacteria received a 12 week oral course of Omnibiotic10AAD^®^. Inguinal swabs and stool samples were collected during and after treatment for microbiological and microbiome analysis. The median age of patients was 76 years. Twelve patients completed the pilot study. Intestinal colonization was reduced to 42% of patients 8 weeks after the end of treatment, but increased to 66% 24 weeks after the end of probiotic treatment. Colonization of inguinal skin was lowest during probiotic treatment and increased thereafter. Fecal microbiome analysis revealed statistically significant increases of the genus *Enterococcus* comparing start and end of probiotic treatment. In conclusion, a 12 week course of a multispecies probiotic led to a transient reduction of intestinal colonization 8 weeks after the end of treatment. The findings of our pilot study warrant further research in the area of probiotics and intestinal colonization by multidrug-resistant bacteria.

## 1. Introduction

Infections due to multidrug-resistant Gram-negative bacteria have become a serious health concern worldwide and lead to elevated health care costs and increased mortality [1,2]. These organisms are capable of colonizing the human gastrointestinal tract, which then constitutes a reservoir in hospitals and in the community [3,4]. Asymptomatic carriage of these organisms carries the risk of subsequent infection, but also implies a potential source of transmission to others, in particular in hospital settings [5,6]. Factors such as fecal incontinence and diarrhea contribute to the subsequent dissemination of pathogens into health care environments. The risk of transmission renders colonization by multidrug-resistant Gram-negative bacteria an important issue in infection control. Several studies have demonstrated that residents in long-term care facilities (LTCFs) are frequently colonized by multidrug-resistant Gram-negative bacteria with rates varying from 4.7% to 64% [6,7,8,9,10].

Efforts to curb gastrointestinal carriage of multidrug-resistant Gram-negatives have included oral administration of nonabsorbable antimicrobial agents such as neomycin and polymyxin E with very limited long-term effects [11,12]. Furthermore, this approach has led to the development of additional antimicrobial resistance in colonizing bacteria [13]. As a consequence, decolonization using antimicrobial agents is not recommended by international societies [14]. Fecal microbiota transplantation (FMT), that is, administration of healthy donor stool into a patient’s gut, has also been studied as a possibility to eradicate multidrug-resistant bacteria from the intestinal tract with varying degrees of success [15,16,17]. However, FMT may not be feasible for all patients affected by intestinal colonization by multidrug-resistant bacteria.

Probiotics are defined as live microbial food supplements with health-promoting attributes when administered in adequate amounts [18]. They have been suggested as a possibility to counteract intestinal colonization by multidrug-resistant bacteria [19]. Several mechanisms of action of probiotics have been proposed, such as (1) colonization of the intestinal tract, preventing colonization by pathogens, a mechanism termed “colonization resistance”; (2) production of toxins and acids to inhibit growth of pathogens; (3) improvement of intestinal barrier function, hence reducing adherence of pathogens to the mucosa; (4) modulation of the host immune system [19,20,21,22].

The aims of our pilot study were to evaluate the effect of the multispecies probiotic Omnibiotic10 AAD^®^ (Institut Allergosan, Graz, Austria) on the intestinal and inguinal skin colonization of patients by multidrug-resistant Gram-negative bacteria in LTCFs in the Geriatric Health Centers Graz, Austria and to evaluate effects of the probiotic on stool and skin microbiome. We chose this particular probiotic formula on the basis of its in vitro characteristics to inhibit growth of intestinal bacteria and to survive a low pH as well as bile [23]. In a study in healthy volunteers taking amoxicillin, intake of this multispecies probiotic led to a significant increase in the mean number of enterococci in stool [23]. Furthermore, clinical observations by one of the authors (M.S.) suggested efficacy of Omnibiotic10 AAD^®^ in eliminating colonization by multidrug-resistant *Acinteobacter baumannii* isolates following an outbreak [24].

## 2. Materials and Methods

### 2.1. Setting

The Geriatric Health Centers Graz is a local institution comprising, among others, an apallic care unit (ACU) caring long term for patients with disorders of consciousness and three long-term geriatric wards. Patients who are treated at the long-term geriatric wards (total of 90 beds) are chronically ill, elderly patients who need constant medical attention.

Upon admission to the ACU and the geriatric wards, all patients are routinely screened for colonization by multidrug-resistant bacteria, including multidrug-resistant Gram-negative bacteria (*Pseudomonas aeruginosa*, *Acinetobacter baumannii*, enterobacteriaceae including extendend sprectrum betalactamase (ESBL)-producers). This initial screening is part of the local infection control strategy. All patients who were colonized by multidrug-resistant Gram-negative (MRGN) bacteria as defined by the Robert Koch Institute, Germany, in the gastrointestinal tract were eligible for the study [25]. Patients were excluded if they had a history of solid organ transplantation or allogeneic hematopoietic stem cell transplantation, an active hematooncological malignancy or neutropenia, acute pancreatitis, or an ongoing immunosuppressive therapy [26]. Demographic data were obtained at study initiation. Collected variables included age, sex, functional status (bedridden or mobile, incontinence), presence of an enteral feeding tube, urinary catheter, comorbidities, and current medication including proton pump inhibitors and any immunosuppressive agents. Informed written consent was obtained from the patients or their legal representatives. The study was approved by the local ethics committee (votum number: 29-569 ex 16/17, Medical University of Graz).

### 2.2. Study Design

All patients received Omnibiotic AAD 10^®^ (Institut Allergosan, Graz, Austria) twice daily orally or via the enteral feeding tube for 3 months. It consists of 10 different bacterial strains: *Enterococcus faecium* W54, *Lactobacillus acidophilus* W55 and W37, *L. paracasei* W72, *L. rhamnosus* W71, *L. salivarius* W24, *L. plantarum* W62, *Bifidobacterium bifidum* W23, *B. lactis* W18, und *B. longum* W51. Patients were screened for side effects during the study by measuring body temperature daily and daily visits by treating physicians looking for signs and symptoms (including nausea, diarrhea, discomfort, loss of appetite, etc.).

**Figure 1:**
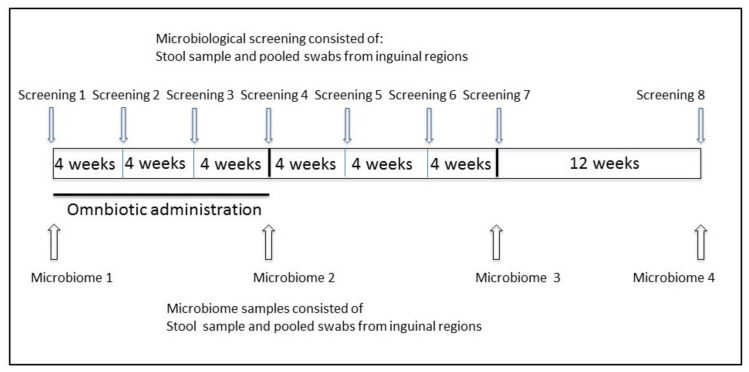
Study timeline.

Inguinal skin swabs were collected by local nursing staff following a standardized procedure. Screening was scheduled in the morning ward round before bathing and dressing the patients. Stool samples were collected by local nursing staff. Both inguinal swabs and stool samples were collected at the start of the study before probiotic treatment was initiated, followed by samples at weeks 4, 8, 12, 16, 20, 24, and 36 (Figure 1). The primary endpoint was successful intestinal decolonization, defined as two consecutive MRGN-negative stool samples at weeks 24 and 36. The secondary endpoint was successful decolonization from skin, defined as two consecutive MRGN-negative skin samples at weeks 24 and 36.

### 2.3. Microbiological Methods

The swabs (Transystem, Copan, Brescia, Italy) and stool samples were transported immediately to the microbiological laboratory of the Section of Infectious Diseases and Tropical Medicine at the Medical University of Graz. Samples were plated on ChromID ESBL^®^ and McConkey agars (bioMerieux, Marcy l’Etoile, France). The plates were incubated under aerobic conditions at 36 °C and were evaluated for growth after 24 and 48 h. Suspected colonies were identified to species level using MALDI-TOF (Bruker, Billerica, MA, USA), and antimicrobial susceptibility was tested by disk diffusion according to European Committee on Antimicrobial Susceptibility Testing (EUCAST) breakpoints. Extended betalactamase (ESBL) production was determined with Clinical and Laboratory Standards Institute (CLSI) confirmatory test using Etest strips (bioMerieux, Marcy l’Etoile, France). Isolates were classified as 3MRGN or 4MRGN according to the Robert Koch Institute guidance document [25]. In brief, isolates resistant to three out of four relevant antimicrobial classes (acylureidopenicillin, third/fourth-generation cephalosporins, carbapenems, fluoroquinolones) were classified as 3MRGN. Enterobacteriaceae resistant to carbapenems were classified as 4MRGN even if the isolate remained susceptible to one other antibiotic class. Isolates resistant to all four classes were classified as 4MRGN. All isolates were stored at −70 °C for analysis of genetic relatedness by whole-genome sequencing at the end of the study. Swabs for skin microbiome analysis (FLOQ Swabs, Copan, Brescia, Italy) and stool samples for microbiome analysis were taken at the same time as microbiological samples and were stored at −70 °C for analysis at the end of the study.

### 2.4. Whole-Genome Sequencing

From each patient, the isolates with identical phenotypes in terms of antimicrobial susceptibility were selected as follows: one isolate from stool at the start of the study, one isolate from stool during the study, and at least one isolate from skin. Table A2 shows all isolates that were selected with their susceptibility patterns. For *E. coli* isolates, genomic DNA isolation, genome library preparation using Nextera XT chemistry (Illumina Inc., San Diego, CA, USA), and whole-genome sequencing using an Illumina MiSeq instrument (Illumina Inc., San Diego, CA, USA) were performed as described previously [27]. Assessment of the core genome multilocus sequence typing (cgMLST) was done using the Enterobase cgMLST scheme (Enterobase.warwick.ac.uk. Available at: http://enterobase.warwick.ac.uk). Assembled genomes were compared using the Enterobase core genome (cg)MLST scheme (http://enterobase.warwick.ac.uk) using SeqSphere+ with a cluster type threshold (CT) of ten allelic differences. For *K. pneumoniae* isolates, extraction of genomic DNA, genomic library preparation, and whole-genome sequencing was performed as described [28]. For phylogenetic analysis, the classical multilocus sequence type (MLST) and the cgMLST were extracted from the whole-genome sequence data [29]. Based on the defined *K. pneumoniae* sensu lato cgMLST in SeqSphere+, comprising 2358 target genes, a gene-by-gene approach was used to compare genomes. Isolates were visualized as minimum spanning trees (MST), and genotypically related isolates were identified with a Complex Type Distance of 15 alleles (https://www.cgmlst.org/ncs/schema/2187931/).

### 2.5. DNA Isolation for Microbiome Analysis, 16S rRNA Gene PCR Amplification and Sequencing

Total DNA was isolated from stool samples as well as dermal swab samples by mechanic and enzymatic lysis according to standard procedures as published in Klymiuk et al. [30]. Briefly, dermal swabs were put into 250 µL PBS (Roth, Karlsruhe, Germany) with 250 µL bacterial lysis buffer (Roche, Mannheim, Germany) and vortexed vigorously. Of the solution, 250 µL was transferred to MagnaLyser tubes (Roche, Mannheim, Germany) and homogenized at 6500 rpm for 30 s, two times each in a MagnaLyser instrument (Roche, Mannheim, Germany). After adding 25 µL lysozyme (100 mg/mL, Roth), samples were incubated at 37 °C for 30 min, treated with proteinase K (20 mg/mL) at 65 °C for 60 min, and incubated at 95 °C for 10 min for inactivation. Total DNA was extracted from 250 µL lysed supernatant on a MagNA Pure LC 2.0 (Roche, Mannheim, Germany) according to manufacturer’s instructions with the MagNA Pure LC DNA Isolation Kit III (Bacteria, Fungi) (Roche, Mannheim, Germany). Similarly, 175 mg of stool sample was homogenized in 500 µL PBS, and 250 µL of the suspensions was used with 250 µ bacterial lysis buffer for DNA isolation as described above. Empty tubes as negative controls were run with each isolation batch. Of total DNA, 2 µL (for stool samples) to 5 µL (for dermal samples) was used in a 25 µL PCR reaction in triplicate using a FastStart High Fidelity PCR system (Roche, Mannheim, Germany) according to Klymiuk et al. with the target specific primers F27-AGAGTTTGATCCTGGCTCAG and R357- CTGCTGCCTYCCGTA [30,31]. The 6 pM library was sequenced on an Illumina MiSeq desktop sequencer (Eindhoven, Netherlands) with 20% PhiX control DNA (Illumina, Eindhoven, Netherlands) and v3 chemistry for 600 cycles in paired-end mode according to manufacturer’s instructions. FastQ raw reads were used for subsequent data analysis. A total of 3,847,966 (stool) and 3,201,263 (dermal swabs) MiSeq paired-end raw sequence forward and reverse reads were quality-filtered, denoised, dereplicated, merged, and checked for chimeras using DADA2 pipeline [32] with standard settings as implemented in QIIME2 2018.7 microbiome bioinformatics platform [33]. Taxonomic assignment of the DADA2 representative sequence set was provided with the QIIME2 sklearn-based classifier against SILVA rRNA database Release 132 at 99% identity [34]. Phylogenetic tree was created with FastTree on Mafft-aligned representative sequences [35,36]. Finally, the DADA2-generated feature table was reduced by removing all features present in only one sample with less than 10 reads (singletons). Further downstream statistical data analysis including alpha and beta diversity was conducted with the R 3.5.1 program for statistical computing (https://www.R-project.org). To calculate the size factors for the normalization of the feature table we used GMPR method, and for the differential taxa analysis between investigated groups count-based regression model DESeq2 was applied [37,38].

## 3. Results

### 3.1. Patients’ Characteristics

Stool samples of 132 patients at the Geriatric Health Centers Graz were screened for multidrug-resistant Gram-negative bacteria. Thirty-two samples were positive. Eleven patients or their legal representatives did not give their consent to participate in the study. Therefore, twenty-one patients were included in the study (median age 76 years, range 19–100; 8/21 female). The mean duration of stay at the wards was 55 ± 55 months, range 1–191. Clinical characteristics are summarized in Table 1. During the study, one patient was transferred to a different long-term care facility and was lost to follow-up. Eight patients died (five after cessation of probiotic treatment). Causes of death were pneumonia (n = 2), aspiration pneumonia (n = 3), heart failure (n = 2), stroke (n = 1). Patient charts were reviewed by two independent investigators. There was no evidence suggestive of a causal relationship between probiotic treatment and death. One patient suffered from loss of appetite as a side effect of probiotic treatment. Twelve patients completed the study. Of those, seven were treated with proton pump inhibitors throughout the study. Of the patients who completed the study, 5/12 received antibiotic treatment (4 with amoxicillin/clavulanate, 1 with cefuroxime; 3 during probiotic treatment, 2 after probiotic treatment). In supplement B, Table 1, patients who received antibiotics are marked.

### 3.2. Prevalence of Colonization by MRGN Bacteria During and after Probiotic Treatment

#### 3.1.1. Stool

**Figure 2:**
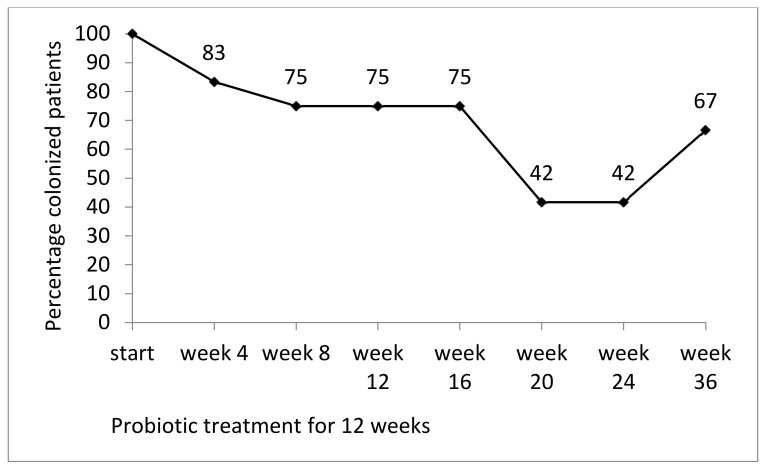
Percentage of patients colonized by multidrug-resistant Gram negative (MRGN) bacteria in stool (n = 12) during and after a 12 week probiotic treatment.

At the beginning of the study, all patients (n = 12, those who completed the study) were colonized by multidrug-resistant Gram negatives: 8/12 by *E. coli*, 3 by *Klebsiella* spp., and 1 by *Pseudomonas aeruginosa*. At the end of the probiotic treatment (week 12), 9/12 patients were still colonized (per protocol analysis, omitting those lost to follow-up). At weeks 20 and 24, only 42% of patients (5/12) were colonized. At week 36, 66% (8/12) were colonized (Figure 2). The primary endpoint (two consecutive MRGN-negative stool samples at weeks 24 and 36) was met by 4/12 patients (4/9 patients from ACU and 0/3 patients from the geriatric wards). A detailed summary of all MRGN isolates of all patients who completed the study is given in Appendix B, Table A1.

#### 3.1.2. Skin

**Figure 3:**
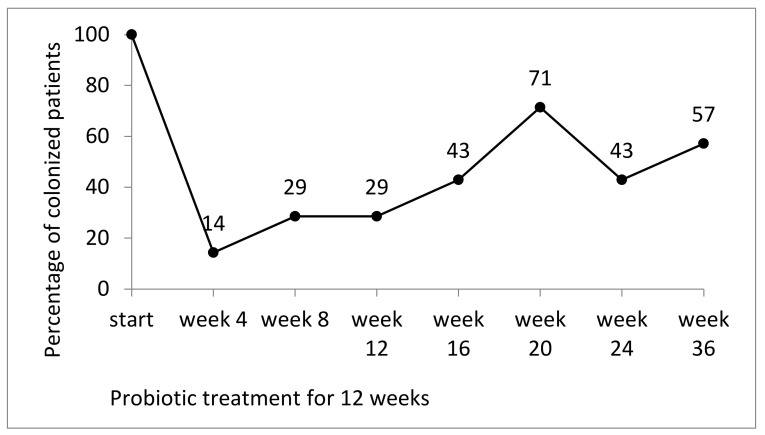
Percentage of patients colonized by MRGN bacteria on skin of the inguinal region (n = 7) during and after a 12 week probiotic treatment.

At the beginning of the study, 7 of 12 patients who completed the study were colonized by multidrug-resistant Gram negatives on their inguinal skin: 2 by *E. coli*, 3 by *Klebsiella* spp., 1 by *Pseudomonas aeruginosa*, and 1 by *Acinetobacter baumannii*. Colonization of the skin in the inguinal regions decreased during probiotic treatment (to 14% at week 4, 28% at weeks 8 and 12). After the end of the treatment period, there was an increase up to 71% at week 20 in the per protocol analysis including the 7 patients who were initially colonized and completed the study (Figure 3). The secondary endpoint (two consecutive MRGN-negative skin samples at week 24 and 36) was met by 3/7 patients (1/5 patients from ACU and 2/2 patients from the geriatric wards).

### 3.3. Genetic Relatedness of E. coli and K. pneumoniae Isolates

**Figure A1:**
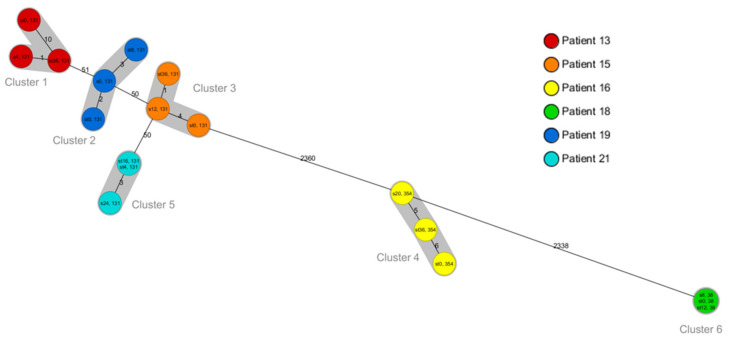
Results of core genome multilocus sequence typing in 18 *E. coli* isolates (cluster distance threshold 10).

**Figure A2:**
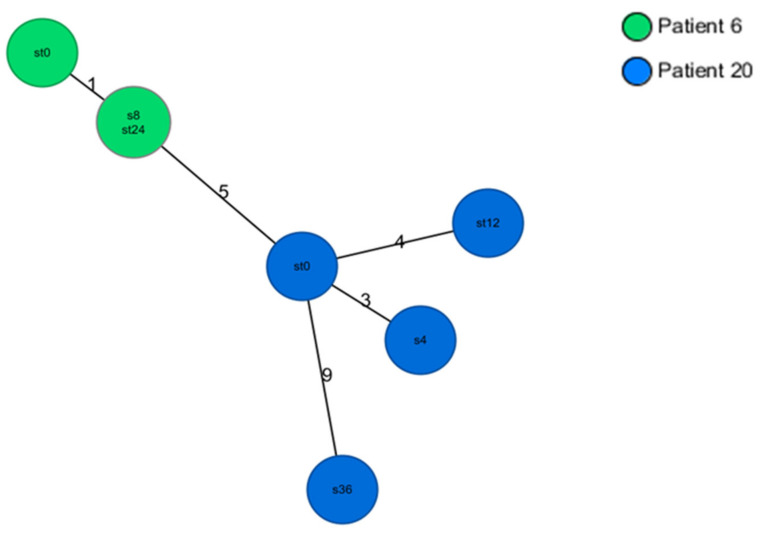
Results of core genome multilocus sequence typing in 7 *K. pneumoniae* isolates (cluster distance threshold 15).

A total of 18 *E. coli* isolates from 6 patients were analyzed by core genome multilocus sequence typing. *E. coli* isolates of four patients were all found to be ST 131. However, isolates from different patients differed in complex type. Isolates from patients 16 and 18 were found to be ST 354 and ST 38, respectively (Appendix A, Figure A1). Each patient was colonized by the same ST and complex type throughout the study period. A total of 7 *K. pneumoniae* isolates from 2 patients were analyzed. *K. pneumoniae* core genome multilocus sequence typing of 7 isolates from 2 patients revealed ST 15 for all isolates. The differences in alleles were below the threshold of 15 alleles (i.e., the isolates were identical). Both patients stayed at the same ward (Appendix A, Figure A2).

### 3.4. Microbiome Analysis

#### 3.4.1. Stool

**Figure 4:**
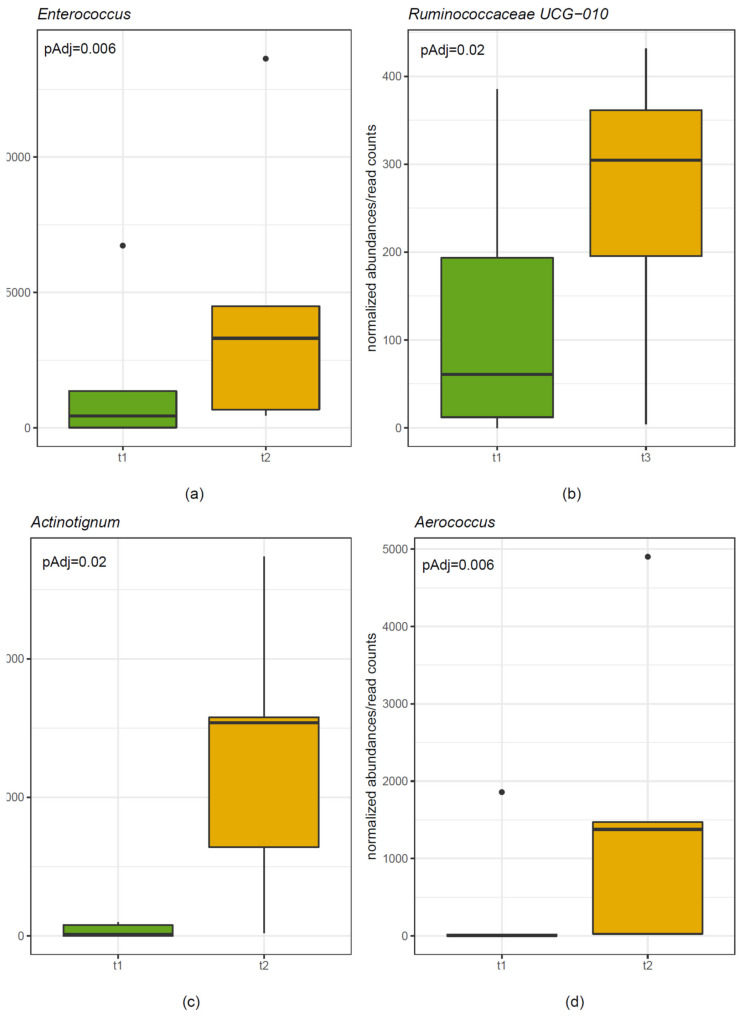
Stool microbiome analysis. (**a**) Statistically significant increase of the genus *Enterococcus* at the end of probiotic treatment (t2) compared with start (t1), *p* = 0.00642. (**b**) Statistically significant increase of the genus *Ruminococcaceae* at week 24 (t3) compared with start (t1), *p* = 0.02. Skin microbiome analysis. (**c**) Statistically significant increase of the genus *Actinotignum* at the end of probiotic treatment (t2) compared with start (t1), *p* = 0.00201. (**d**) Statistically significant increase of the genus *Aerococcus* at the end of probiotic treatment (t2) compared with start (t1), *p* = 0.005654.

The stool microbiome of five randomly selected patients was analyzed at four different time points (start before probiotic treatment, week 12 = end of probiotic treatment, week 24, and week 36). There was no significant difference in alpha-diversity (Shannon Diversity Index) and beta-diversity (Weighted UniFrac distances) comparing different time points (i.e., start, end of treatment, weeks 24 and 36). In-depth analysis revealed statistically significant increases of the genera *Enterococcus* and *Tyzzerella* (Figure 4) comparing start and week 12 (*p* = 0.006 and *p* = 0.0008, respectively). There was a statistically significant increase of the genus *Ruminococcus* comparing start with week 24 (adjusted *p* = 0.02) and of the family Actinomycetaceae comparing start with week 36 (adjusted *p* = 0.02).

#### 3.4.2. Skin

Swabs were taken from the inguinal region of the skin. The skin microbiome of the same randomly selected five patients was analyzed at four different time points (start before probiotic treatment, week 12 = end of probiotic treatment, week 24, und week 36). There was no significant difference in alpha-diversity (Shannon Diversity Index) and beta-diversity (Weighted UniFrac distances) comparing different time points. In-depth analysis revealed statistically significant increases of the genera *Aerococcus* and *Actinotignum* (Figure 4) comparing start and week 12 (adjusted *p* = 0.005 and *p* = 0.002, respectively). In addition, there were significant increases of the order Actinomycetales and the family Actinomycetacae of which *Actinotignum* is a part (adjusted *p* = 0.006 and *p* = 0.004) comparing start and week 12.

## 4. Discussion

This pilot study aimed to assess the effect of the multispecies probiotic Omnibiotic AAD 10^®^ on colonization by multidrug-resistant Gram-negative bacteria in patients in LTCFs. Notable findings were (1) intestinal colonization was reduced to 42% of patients 8 weeks after the end of treatment (week 20); (2) fecal microbiome analysis revealed statistically significant increases of the genus *Enterococcus* comparing start and week 12 (end of probiotic treatment); (3) colonization of inguinal skin was lowest during probiotic treatment.

We expected a decrease in the intestinal colonization during probiotic treatment. However, the prevalence of intestinal colonization by multidrug-resistant Gram-negative bacteria decreased only to 75% at the end of probiotic treatment at week 12, followed by a marked reduction to 42% 8 and 12 weeks after the end of treatment (i.e., weeks 20 and 24). Fecal microbiome analysis revealed a significant increase in the genus *Ruminococcaceae* at week 24. In contrast, the genus *Enterococcus* significantly increased at the end probiotic treatment (week 12) but not later on. *Enterococcus faecium* was a component of the multispecies probiotic used in the study. Our findings suggest that even the transient changes of the fecal microbiome may influence colonization by MRGN bacteria. Even though the probiotic strains *Lactobacillus rhamnosus* and *L. paracasei* inhibited the growth of various Gram-negative bacteria, including *E.coli*, *Klebsiella* spp. and *P. aeruginosa,* in vitro [39], direct antimicrobial activity of probiotic strains, such as production of toxins or acids to inhibit the growth of other bacteria, are less likely to have played a role in our study.

At week 36, two patients who were negative for MRGN bacteria at weeks 20 and 24 were positive for MRGN bacteria that had not been detected before in these patients. This finding suggests a new colonization from the environment of the LTCF. One could speculate that a combination of repeated probiotic treatment and enforced infection control could be a successful strategy for maintaining a decolonized status.

Ljungquist et al. recently published a placebo-controlled clinical trial using a different multispecies probiotic (Vivomixx^®^) in patients with intestinal colonization by ESBL-producing enterobacteria [40]. Successful intestinal decolonization was achieved only in 12.5% in the probiotics group compared with 5% in the control group, a difference that was not statistically significant. The probiotic used in the study consisted of *Lactobacillus* spp., *Bifidobacterium* spp., and *Streptococcus thermophiles,* according to the producers. The probiotic used in our study also contained *Lactobacillus* spp. and *Bifidobacterium* spp. but in addition, *E. faecium*, a species that was found to have increased significantly in numbers at the end of the treatment (week 12) in the microbiome analysis. Results on the potential for intestinal decolonization cannot be generalized between different probiotics.

We chose to study inguinal swabs as inguinal colonization is thought to have a high impact on cross-transmission in health care settings and is of importance in terms of infection control [6]. Inguinal skin is assumed to be colonized by MRGN bacteria from the intestinal tract, particularly in patients suffering from fecal incontinence. This assumption is supported by the detection of genetically identical *E. coli* and *K. pneumoniae* isolates from stool and inguinal skin of the same patient in our study. A possibly favorable effect of oral probiotics on skin colonization could unfold by similar mechanisms. However, recent evidence suggests that the intestinal microbiota and their metabolites also impact cutaneous physiology and immune response in a complex fashion via the “gut–skin axis” [41].

Probiotics have been demonstrated to exert beneficial effects on skin health [42,43,44]. For example, a two month supplementation of *Lactobacillus paracasei* decreased skin sensitivity and increased the rate of barrier function recovery in a placebo-controlled trial [43]. In our study, the prevalence of inguinal colonization by MRGN bacteria was lowest during probiotic treatment and increased steadily thereafter. As most patients were colonized by various different bacteria in the inguinal region that were not found in their intestinal tract, recolonization from the environment is likely to have occurred in addition to colonization from the intestinal tract. Significant increases of the taxa *Aerococcus* and *Actinotignum* were detected in skin microbiome at the end of probiotic treatment (week 12) but not later on. This could suggest that probiotic strains led to a transient change in skin microbiome which, in turn, reduced colonization by MRGN Gram-negative bacteria.

Intestinal and skin colonization by MRGN *K. pneumoniae* and *E. coli* persisted throughout the study period in serval patients. For example, patient 16 was colonized by genetically identical *E. coli* isolates at the start of the study and at week 36. This is in line with data from a meta-analysis from 2016 which found that a significant proportion of carriers of ESBL-producing enterobacteria remained colonized for up to one year in health care settings [45]. Two patients (6 and 20) staying at the same ward were colonized by genetically identical *K. pneumoniae* isolates. This finding could indicate cross-contamination between patients by staff may have taken place, as has been described before [24]. Due to financial constraints, not all isolates could be analyzed by whole genome sequencing. Selection of the isolates was based on susceptibility testing patterns, which may have led to selection bias.

In our study, the probiotic was safe and tolerated. Side effects (loss of appetite) occurred only in one patient. This is in accordance to a recent meta-analysis that found probiotics to be safe in the elderly [46]. However, it should be kept in mind that patients with active hematooncological malignancy, neutropenia, acute pancreatitis, or an ongoing immunosuppressive therapy were excluded from the study.

This study has several strengths. Adherence to the interventional product was very high because all patients stayed at a LTCF during the study period. Microbiological testing was performed frequently, that is, once a month until week 24, followed by a final examination at week 36. The major limitations of the study are the lack of a placebo-controlled design and the limited number of participants, which are limitations owing to the design as a pilot study. However, our findings justify future research on the effects of the multispecies probiotic Omnibiotic AAD 10^®^ on intestinal colonization. In addition, we only included patients from LTFCs in our study. These patients are likely to be different in many physiologic aspects compared with elderly persons living at home. Our findings may not be generalizable to other groups of patients. Furthermore, none of the patients who finished the trial experienced infections due to multidrug-resistant Gram-negative bacteria. In conclusion, a 12 week course of the multispecies probiotic Omnibiotic AAD 10^®^ led to a reduction of intestinal colonization 8 weeks after the end of treatment. Our findings warrant further research in the area of probiotics and intestinal colonization by MRGN bacteria.

## Figures and Tables

**Table 1:** Clinical characteristics of all 21 patients enrolled.

Characteristics	ACU	Geriatric Wards
Number of patients	12	9
Age (years, median, range)	59 (19–80)	86 (77–100)
Gender, n (%)		
Male	8 (66)	5 (55)
Female	4 (33)	4 (45)
Comorbidities, n (%)		
Parkinson’s disease	0	2 (22)
Coronary heart disease	0	2 (22)
Dementia	0	7 (77)
Functional status, n (%)		
Bedridden	12 (100)	2 (22)
Bowel incontinence, n (%)	12 (100)	6 (66)
Bladder incontinence, n (%)	12 (100)	4 (45)
Gastrostomy, n (%)	12 (100)	1 (11)
Antibiotic treatment, n (%)		
During probiotic	5 (41)	0
After probiotics	3 (25)	0

ACU = apallic care unit.

## Appendix B

**Table A1:** Antimicrobial susceptibility of MRGN isolates that were selected for whole genome sequencing

Isolate	Amox/clav	Pip	Pip/Taz	CAZ	Cefepim	Mero	Imi	Cipro	Azt	Genta	ESBL
Patient 6											
*K. pneumoniae* (week 0, stool)	R	R	S	R	R	S	S	R	R	R	+
*K. pneumoniae* (week 24, stool)	R	R	S	R	R	S	S	R	R	R	+
*K. pneumoniae* (week 8, skin)	R	R	S	R	R	S	S	R	R	R	+
Patient 20											
*K. pneumoniae* (week 0, stool)	R	R	S	R	R	S	S	R	R	R	+
*K. pneumoniae* (week 12, stool)	R	R	S	R	R	S	S	R	R	R	+
*K. pneumoniae* (week 4, skin)	R	R	S	R	R	S	S	R	R	R	+
*K. pneumoniae* (week 36, skin)	R	R	S	R	R	S	S	R	R	R	+
Patient 15											
*E. coli* (week 0, stool)	R	R	S	R	R	S	S	R	R	S	+
*E. coli* (week 36, stool)	R	R	S	R	R	S	S	R	R	S	+
*E. coli* (week 12, skin)	R	R	S	R	R	S	S	R	R	S	+
Patient 19											
*E. coli* (week 0, stool)	R	R	S	R	R	S	S	R	R	S	+
*E. coli* (week 8, stool)	R	R	S	R	R	S	S	R	R	S	+
*E. coli* (week 0, skin)	R	R	S	R	R	S	S	R	R	S	+
Patient 13											
*E. coli* (week 0, stool)	R	R	S	R	R	S	S	R	R	R	+
*E. coli* (week 36, stool)	R	R	S	R	R	S	S	R	R	R	+
*E. coli* (week 4, skin)	R	R	S	R	R	S	S	R	R	R	+
Patient 16											
*E. coli* (week 0, stool)	R	R	S	R	R	S	S	R	R	R	+
*E. coli* (week 36, stool)	R	R	S	R	R	S	S	R	R	R	+
*E. coli* (week 20, skin)	R	R	S	R	R	S	S	R	R	R	+
Patient 18											
*E. coli* (week 0, stool)	R	R	S	R	R	S	S	R	R	R	+
*E. coli* (week 12, stool)	R	R	S	R	R	S	S	R	R	R	+
*E. coli* (week 8, skin)	R	R	S	R	R	S	S	R	R	R	+
Patient 21											
*E. coli* (week 4, stool)	R	R	S	R	R	S	S	R	R	R	+
*E. coli* (week 16, stool)	R	R	S	R	R	S	S	R	R	R	+
*E. coli* (week 24, skin)	R	R	S	R	R	S	S	R	R	R	+

E. coli = *Escherichia coli*; K. pneumoniae = *Klebsiella pneumonia*, S = susceptible, R= resistant according to EUCAST. Amox/clav = amoxicillin/clavulanate; Pip = piperacillin, Pip/Taz = piperacillin/tazobactam; CAZ = ceftazidime; Mero = meropenem; Imi = imipenem; Cipro = ciprofloxacin; Azt = aztreonam; Genta = gentamicin, ESBL = extended betalactamase.

**Table A2:** Colonization by 3MRGN or 4MRGN bacteria in stool and inguinal skin of 12 patients who completed the study.

Patient		Start	Week 4	Week 8	Week 12	Week 16	Week 20	Week 24	Week 36
1	Stool	*E. coli*	*-*	*-*	*-*	*-*	*-*	*-*	*-*
	Skin	*A. baumannii*	*-*	*-*	*-*	*-*	*A. baumannii*	*A. baumannii*	*A. baumannii*
2	Stool	*K. oxytoca*	*-*	*K. oxytoca* ^AC^	*K. oxytoca*	*-*	*-*	*-*	*-*
	Skin	*-*	*-*	*K. oxytoca*	*K. oxytoca*	*-*	*-*	*-*	*A. baumanni*
5	Stool	*E. coli*	*E. coli/ K. aerog.*	*E. coli*	*E. coli*	*E. coli*	*-*	*E. coli*	*E. coli*
	Skin	*E. cloacae*	*-*	*-*	*-*	*E. coli*	*E. coli*	*-*	*-*
6	Stool	*K. pneumoniae* ^#^	*K. pneumoniae*	*K. pneumoniae* ^C^	*-*	*K. pneumoniae*	*K. pneumoniae*	*K. pneumoniae* ^#^	*K. pneumoniae*
	Skin	*-*	*K. pneumoniae*	*K. pneumoniae* ^#^	*K. pneumoniae*	*K. pneumoniae*	*-*	*-*	*-*
12	Stool	*P. aeruginosa*	*P. aeruginosa*	*P. aeruginosa*	*P. aeruginosa*	*P. aeruginosa*	*-*	*-*	*-*
	Skin	*-*	*-*	*-*	*-*	*-*	*-*	*-*	*-*
13	Stool	*E. coli* *	*E. coli*	*-*	*E. coli*	*E. coli*	*-*	*E. coli*	*E. coli**
	Skin	*-*	*E. coli* *	*E. coli*	*E. coli*	*-*	*E. coli*	*E. coli*	*-*
15	Stool	*E. coli* ^§^	*-*	*E. coli*	*E. coli*	*E. coli*	*E. coli*	*E. coli*	*E. coli* ^§^
	Skin	*E. coli*	*-*	*-*	*E. coli^§^*	*-*	*E. coli*	*-*	*-*
16	Stool	*E. coli* ^&^	*E. coli*	*E. coli*	*E. coli*	*E. coli*	*E. coli*	*E. coli*	*E. coli* ^&^
	Skin	*K. pneumoniae*	*-*	*K. pneumoniae*	*K. pneumoniae*	*K. pneumoniae*	*E.coli^&^/K. pn.*	*-*	*K. pneumoniae*
18	Stool	*E. coli* ^$^	*E. coli* ^AC^	*-*	*E. coli* ^$^	*A. baumannii*	*-*	*-*	*M. morganii*
	Skin	*P. aeruginosa*	*A. baumannii*	*E. coli^$^*	*-*	*A. baumannii*	*A. baumannii*	*A. baumannii*	*P. aeruginosa*
19	Stool	*E. coli* °	*E. coli*	*E. coli°*	*-*	*-*	*-*	*-*	*M. morganii*
	Skin	*E. coli* °	*-*	*-*	*-*	*-*	*-*	*-*	*-*
20	Stool	*K. pneumoniae* ^#^	*K. pneumoniae*	*K. pneumoniae*	*K. pneumoniae* ^#^	*K. pneumoniae* ^AC^	*K. pneumoniae*	*-*	*-*
	Skin	*-*	*K. pneumoniae* ^#^	*K. pneumoniae*	*K. pneumoniae*	*K. pneumoniae*	*K. pneumoniae*	*-*	*K. pneumoniae* ^#^
21	Stool	*E. coli*	*E. coli* **	*E. coli*	*E. coli*	*E. coli* **	*E. coli*	*-^AC^*	*K. pneumoniae*
	Skin	*E. coli*	*-*	*E. coli*	*-*	*-*	*-*	*E. coli* **	*E. coli*

E. coli = Escherichia coli; A. baumannii = Acinetobacter baumannii, K. aerog. = Klebsiella aerogenes; K. oxytoca = Klebsiella oxytoca; K. pn. = K. pneumoniae = Klebsiella pneumoniae; E. cloacae = Enterobacter cloacae; M. morganii = Morganella morganii. ^#^ These K. pneumoniae isolates were sequence type 15 belonging to the same cluster.^AC^ = amoxicillin/clavulanate, ^C^ = cefuroxime. * All isolates were sequence type 131 belonging to cluster 1. ** All isolates were sequence type 131 belonging to cluster 5. ^§^ All isolates were sequence type 131 belonging to cluster 3. ° All isolates were sequence type 131 belonging to cluster 2. ^&^ All isolates were sequence type 354. ^$^ All isolates were sequence type 38. Isolates of patients who did not complete the study are not shown.
